# Mevalonate-Farnesal Biosynthesis in Ticks: Comparative Synganglion Transcriptomics and a New Perspective

**DOI:** 10.1371/journal.pone.0141084

**Published:** 2016-03-09

**Authors:** Jiwei Zhu, Sayed M. Khalil, Robert D. Mitchell, Brooke W. Bissinger, Noble Egekwu, Daniel E. Sonenshine, R. Michael Roe

**Affiliations:** 1 Department of Entomology, North Carolina State University, Raleigh, North Carolina, 27695, United States of America; 2 Department of Biological Sciences, Old Dominion University, Norfolk, Virginia, 23529, United States of America; University of Maryland, College Park, UNITED STATES

## Abstract

Juvenile hormone (JH) controls the growth, development, metamorphosis, and reproduction of insects. For many years, the general assumption has been that JH regulates tick and other acarine development and reproduction the same as in insects. Although researchers have not been able to find the common insect JHs in hard and soft tick species and JH applications appear to have no effect on tick development, it is difficult to prove the negative or to determine whether precursors to JH are made in ticks. The tick synganglion contains regions which are homologous to the corpora allata, the biosynthetic source for JH in insects. Next-gen sequencing of the tick synganglion transcriptome was conducted separately in adults of the American dog tick, *Dermacentor variabilis*, the deer tick, *Ixodes scapularis*, and the relapsing fever tick, *Ornithodoros turicata* as a new approach to determine whether ticks can make JH or a JH precursor. All of the enzymes that make up the mevalonate pathway from acetyl-CoA to farnesyl diphosphate (acetoacetyl-CoA thiolase, HMG-S, HMG-R, mevalonate kinase, phosphomevalonate kinase, diphosphomevalonate decarboxylase, and farnesyl diphosphate synthase) were found in at least one of the ticks studied but most were found in all three species. Sequence analysis of the last enzyme in the mevalonate pathway, farnesyl diphosphate synthase, demonstrated conservation of the seven prenyltransferase regions and the aspartate rich motifs within those regions typical of this enzyme. In the JH branch from farnesyl diphosphate to JH III, we found a putative farnesol oxidase used for the conversion of farnesol to farnesal in the synganglion transcriptome of *I*. *scapularis* and *D*. *variabilis*. Methyltransferases (MTs) that add a methyl group to farnesoic acid to make methyl farnesoate were present in all of the ticks studied with similarities as high as 36% at the amino acid level to insect JH acid methyltransferase (JHAMT). However, when the tick MTs were compared to the known insect JHAMTs from several insect species at the amino acid level, the former lacked the farnesoic acid binding motif typical in insects. The P450s shown in insects to add the C10,11 epoxide to methyl farnesoate, are in the CYP15 family; this family was absent in our tick transcriptomes and in the *I*. *scapularis* genome, the only tick genome available. These data suggest that ticks do not synthesize JH III but have the mevalonate pathway and may produce a JH III precursor.

## Introduction

Ticks are ectoparasites and important vectors of human and animal diseases. They ranked second only to mosquitoes as vectors of life-threatening or debilitating human and animal diseases and transmit a larger variety of pathogen-borne diseases than any other arthropod [1]. Pathogens harbored by ticks cause Lyme disease, Rocky Mountain spotted fever, tick paralysis, tick toxicoses, heartwater disease, irritation, tick bite allergies, immune responses and others diseases and cause economic losses in animal production due to blood loss and disease. How ticks regulate their development and reproduction is of special interest because of their unique life style as obligatory blood feeders and their ancient divergence from crustaceans and insects [2]. Also, understanding the endocrinology of ticks like that in insects will provide new approaches to their control.

Insect molting, development through instars and stages, metamorphosis and reproduction are regulated by two hormones, ecdysteroids and juvenile hormone (JH). The presence of ecdysteroids and JH at the same time produces a larval-larval molt, ecdysteroids in the absence of JH initiate metamorphosis, and in most insects studied, JH initiates the synthesis and deposition of yolk protein in the eggs, a process known as vitellogenesis [3–6]. Crustacea (also a mandibulate group like insects) use ecdysteroids and methyl farnesoate but not JH, to regulate their development [6–12].

Because ticks and mites as terrestrial arthropods are similar in appearance and have similar developmental stages as insects, the general assumption for many years has been that ticks were like insects in the hormones used for regulating reproduction [1]. This was especially supported by Pound and Oliver [13] in studies with ticks and Oliver et al. [14] in mites where they found that JH *in vivo* could rescue anti-JH effects and initiate egg development. However, since these initial reports, a number of subsequent studies have not supported a role for JH in egg development in ticks. Taylor et al. [15] and Chinzei et al. [16] found JH topically applied to ticks did not affect egg production; Sankhon et al. [17] showed that ecdysteroids initiated vitellogenin synthesis in fat body in organ culture; Friesen and Kaufman [18] showed that ecdysteroids did the same *in vivo*; and Thompson et al. [19, 20] and Khalil et al. [21] showed that ecdysteroids and not JH *in vivo* resulted in the expression of the vitellogenin (Vg) message, the appearance of Vg protein in the hemolymph, and the production of brown (vitellogenic) eggs. In addition, Neese et al. [22] were unable to find any of the common insect JHs in a species of both hard and soft ticks by highly sensitive radiometric biosynthesis and EI SI GC/MS.

The synthesis of JH III in insects occurs in the stomatogastric nervous system (in the insect head) and consists of two parts, the mevalonate pathway from acetate to farnesyl pyrophosphate followed by the JH branch ending in JH III. There are two well characterized enzymes in the JH branch shown to be involved in JH III biosynthesis in insects, i.e., a JH methyltransferase which adds a methyl ester to produce methyl farnesoate or JH III and a P450 in the family CYP15 which adds a C10*R*,11 epoxide [1, 23]. The characterization of the genes, messages or enzymes in the mevalonate pathway and JH branch, fundamental to the developmental biology of crustaceans and insects, has never been investigated in ticks. With the advancement in high throughput, highly repetitive DNA sequencing and bioinformatics, a tick transcriptome can be examined for the presence or absence of every enzyme in the mevalonate-JH pathway. In the current study, this approach was used to examine separate synganglion transcriptomes of two hard tick species and one soft tick species with comparative work with the first tick genome to provide evidence whether there is a potential role for the mevalonate and at least parts of the JH branch in tick development.

## Materials and Methods

### Ticks

American dog ticks, *Dermacentor variabilis*, were reared as described by Sonenshine [24] and were colonized at the Old Dominion University Animal Facility in Norfolk, VA, USA. The colony originated from ticks originally collected near Richmond, Virginia, USA. Immature stages were fed on Norway rats, *Rattus norvegicus*. Adult male and female ticks were allowed to feed and mate naturally within a plastic capsule attached to their host, the New Zealand white rabbit, *Oryctolagus cuniculus*, or withheld from blood feeding and/or mating depending on the conditions of the particular experiment. Black-legged ticks, *Ixodes scapularis*, were reared as described also by Sonenshine [24]. The ticks were collected near Armonk, NY, USA. Adult ticks were confined within plastic capsules attached to New Zealand white rabbits and allowed to feed to repletion. Larvae and nymphs were allowed to feed on albino mice, *Mus musculus*. Relapsing fever ticks, *Ornithodoros turicata*, were obtained originally from Dr. James H. Oliver, Georgia Southern University, Statesboro, GA, USA. This particular colony originated from burrows of the gopher tortoise in FL, USA. They were maintained in wood chip litter. Adults and nymphs were fed on albino mice. The rearing conditions were 26 ±1°C, 92 ± 1% relative humidity, and 14:10 light versus dark for all ticks.

The animals used for tick rearing were housed in cage sizes in strict accordance with the recommendations in the Guide for the Care and Use of Laboratory Animals of the National Institutes of Health. The animals were provided ad libitum water and a commercial food appropriate to the animal species and animal age. All rodents received at least one housing type enrichment item during cage changes (examples were polycarbonate tunnels (for rats), igloos (for mice), alfa twist or envirodry); all rodents received at least one chewing type enrichment item during cage changes (examples were Nylabone and wooden blocks); and other food enrichment (ex. included but was not limited to sunflower seeds, Fruity Gems®, Vegie Bites® and Fruity Bites®). All rabbits received at least one enrichment item per week unless otherwise directed by the investigator, attending veterinarian or designee, or the facility manager. Examples of items to be given included but was not limited to Nylabones, Bunny Blocks, Jingle Balls and Dumb Bells. All rabbits received food enrichment at least three times per week. Examples of food enrichment included but was not limited to Timothy cubes, hay, Vegie Bites®, Fruity Gems®, and fresh fruit or vegetables.

### Ethics statement

In this study, we strictly followed the recommendations in the Guide for the Care and Use of Laboratory Animals of the National Institutes of Health to minimize pain and discomfort of the animals. The protocols were approved by the Old Dominion University Institutional Animal Care and Use Committee (Animal Welfare Assurance Number: A3172-01). These protocols (#10–018 and #10–032) are on file at the Office of Research, Old Dominion University, Norfolk, VA, USA.

### Tissue dissection, total RNA isolation, cDNA synthesis and sequencing

Synganglia from different adult ticks under study were isolated by dissection and included the pedal nerves and lateral segmental organs. All lab bench surfaces, petri dish dissecting containers and instruments used in the dissections were treated with RNaseZap (Ambion, Austin, TX, USA) to limit RNA degradation. All containers used were autoclaved and kept sterile until needed and never re-used. Because of the challenges of establishing and maintaining three different tick species in the laboratory, generating the number of ticks needed to collect enough tissue for transcriptome construction, developing different aged adults which was dependent in part on the developmental biology which differed between different tick species, the dissection of large numbers of synganglia from different species and different developmental stages, and the need to obtain RNA that was not degraded, the transcriptomes in this study were generated over an extended time period. During this period, there was a rapid evolution in the sequencing and bioinformatics technologies available in our core facility at North Carolina State University. Also for the sake of time and cost, it was not always possible or necessary to standardized all aspects of the transcriptome construction and data analysis to achieve the goals of our research. The 454 and Illumina sequencing was conducted in the Genome Sequencing Laboratory at North Carolina State University.

#### RNA purity

The quality and purity (260/280 nm) of the total RNA was first determined using a Nanodrop 2000 spectrophotometer (Thermofisher, Wilmington, DE, USA). Samples with low purity (<1.8) were discarded. A Bioanalyzer 2100 (Agilent Technologies) was used next to determine the integrity of RNA samples; samples that did not meet minimum requirements (RNA integrity ≥ 8) were discarded [25].

#### *D*. *variabilis* transcriptome

The *D*. *variabilis* synganglion 454 transcriptome was made from approximately 50 synganglia each from unfed, part-fed virgin (attached to the host for 4–5 days), part-fed virgin forcibly detached from the rabbit host and held in culture for 4–5 days, part-fed mated (allowed to mate for 1 day), and replete (1 day post drop off from the host) females. Tissues from each feeding stage were homogenized separately in TRI Reagent (Sigma-Aldrich, St. Louis, MO, USA). RNA pellets were rehydrated in 100 mM aurintricarboxylic acid to prevent degradation [26]. Approximately 3μg total RNA from each group was pooled. The mRNA was isolated using an Oligotex mRNA isolation kit (Qiagen, Valencia, CA, USA). Purified mRNA was precipitated using ethanol then rehydrated and combined with 10 pmol of modified 3′ reverse transcription primer (5′ATTCTAGAGACCGAG GCGGCCGA CATG T(4)GT(9)CT(10) VN-3′) [27] and 10 pmol SMART IV oligo (5′-AAGCAGTGGTATCAACGC AGAGTGGCCATTACGGCCGGG-3′) [28]. The mRNA and primers were incubated at 72°C for 2 min and then combined with the following reagents on ice: 1 ml RnaseOut (40 U/μl), 2 μl 5× first strand buffer, 1 μl 20 mM dithiothreitol (DTT), 1 μl deoxynucleotide triphosphate (dNTP) mix (10 mM each) and 1 μl Superscript II reverse transcriptase (Invitrogen, Carlsbad, CA, USA). The reaction was incubated at 42°C for 90 min, then diluted to 30 μl with Tris-EDTA (TE) buffer (10 mM Tris HCl, pH 7.5, 1 mM ethylenediaminetetraacetic acid) and stored at -20°C until further use. Second strand cDNA was synthesized by combining 5 μl first-strand cDNA with 10 pmol modified 3′ PCR primer (5′ATTCTAGAGGCCGAGGCGGCCGACAT G(4)GTCT(4)GTTCTGT(3)CT(4)VN-3′) [27], 10 pmol of 5′ PCR primer (5′AAGCAGT GGTATCAACGCAGAGT-3′) [28], 5 μl 10× reaction buffer, 1 μl dNTP mix, 2 μl MgSO_4_, 0.4 μl Platinum Taq DNA polymerase High Fidelity and 34.6 μl H_2_O (Invitrogen). Thermal cycling was conducted as follows: 94°C for 2 min followed by 25 cycles of 94°C for 20s, 65°C for 20 s and 68°C for 6 min. The first PCR reaction was conducted, and 5 μl aliquots from cycles 18, 22 and 25 were analyzed on a 1% agarose gel to optimize the number of cycles. Five additional reactions were conducted to produce sufficient quantities of cDNA for 454 library preparation. The contents were combined, and the cDNA was purified using a PCR purification kit (Qiagen) according to the manufacturer’s recommendations. The cDNA library was prepared with appropriate kits (Roche, Indianapolis, IN, USA; Qiagen) for pyrosequencing on the GS-FLX sequencer (Roche) according to the manufacturer’s recommendations described previously by Margulies et al. [29]. The only deviation from the protocol was prior to titration sequencing where following emulsification PCR, DNA-positive beads were enriched to increase the number of reads collected during titration [30].

#### *I*. *scapularis* transcriptomes

A 454 and two different Illumina synganglion transcriptomes were made from adults of *I*. *scapularis*. For the 454 pyrosequencing, tissue processing, RNA extraction and sequencing were conducted as described earlier for the *D*. *variabilis* synganglion transcriptome and also described by Donohue et al. [30] and Egekwu et al. [31]. The dissections of *I*. *scapularis* ticks for the Illumina transcriptomes were conducted in phosphate-buffered saline on ice (pH 7.0, 10mM NaH_2_PO_4_, 14 mM Na_2_HPO_4_, and 150 mM NaCl). The synganglia were homogenized in Qiagen RLT buffer and total RNA extracted following the manufacturer's recommendations (Qiagen). RNA samples were stored at -80°C until needed. The total RNA was 3.25 μg from 30 part-fed virgin females synganglia for 454 pyrosequencing, 5.1 μg from a mixture of 50 unfed, part-fed virgin and replete female synganglia for the first Illumina transcriptome (**I**) and 3.28 μg from 45 part-fed virgin females for the second (**II**) Illumina transcriptome.

For Illumina sequencing, the Illumina TruSeq RNA Sample Prep Kit v2 (Part No. 15026495, Illumina, Inc. San Diego, CA, USA) was used. Following PCR amplification, adapters were included for sequencing with paired ends. For paired ends, Illumina GA-II sequencing adapters were ligated to the fragments as described by the Illumina’s Paired-End Sample Preparation Guide (catalogue number PE-930-1001).

#### *O*. *turicata* transcriptome

For the *O*. *turicata* Illumina transcriptome, 50 synganglia from replete female adult ticks were dissected in phosphate-buffered saline (described earlier) and the synganglia homogenized in Qiagen RLT buffer. Total RNA was extracted according to the manufacturer’s protocol using the RNeasy mini kit (Qiagen). A total of 6.97μg of total RNA was obtained and sequencing conducted using the Illumina Truseq RNA Sample Prep Kit v2 (Part No. 15026495, Illumina). After RT-PCR amplification, paired end sequencing was conducted using Illumina GA-II sequencing adapters ligated to the fragments as described by the Illumina's Paired-End Sample Preparation Guide (catalogue number PE-930-1001) and Egekwu et al. [32].

### Bioinformatics

For each transcriptome, duplicate reads, primer sequence contamination, adapter sequences and ambiguous base calls were trimmed. The assembly of the *D*. *variabilis* synganglion 454 transcriptome was done with GS Assembler (Roche) using default parameters. The other three transcriptomes (*I*. *scapularis* 454; Illumina transcriptome and *O*. *turicata* Illumina transcriptome) were assembled with the CLC-BIO program [33]. The assembled contiguous sequences will be referenced in this study as "contigs." The putative functions of contigs in each transcriptome were identified (annotated) by the Basic Local Alignment Search Tool (BLAST) against the GenBank non redundant database and EST database [34] with an e-value of E-06 (or lower) for the Illumina assemblies and E-10 for 454 pyrosequencing. Additional BLAST searches were done for selected contigs of interest against the conspecific *I*. *scapularis* genome (www.vectorbase.org).

BLASTx and BLASTp searches of the *D*. *variabilis*, *I*. *scapularis* and *O*. *turicata* synganglion transcriptomes as well as transcriptomes generated for the *D*. *variabilis* male reproductive system and midgut described elsewhere [35, 36] were conducted for insect specific matches using the insect enzymes described before in JH III biosynthesis by Noriega et al. [37] and Belles et al. [38]. BLASTp searches were also conducted of the *I*. *scapularis* genome (www.vectorbase.com) using the CYP15A1 message from *D*. *punctata* (AAS13464) proven to catalyze the formation of the C10,11 epoxide of JH [23]. The search revealed 500 CYP genes in the *I*. *scapularis* genome; 20 selected matches based on being full length and having the lowest e-value were utilized to construct an optimal neighbor joining phylogenic tree using the Molecular Evolutionary Genetics Analysis (MEGA) program [39]. Sequence alignments of CYP15A1 were performed with ClustalW (www.ebi.ac.uk/clustalw). Sequence alignments of farnesyl diphosphate synthase, methyltransferase and farnesol oxidase were performed with Jalview Version 2.8.2 [40].

## Results and Discussion

### Tick transcriptomes

**Fig 1:**
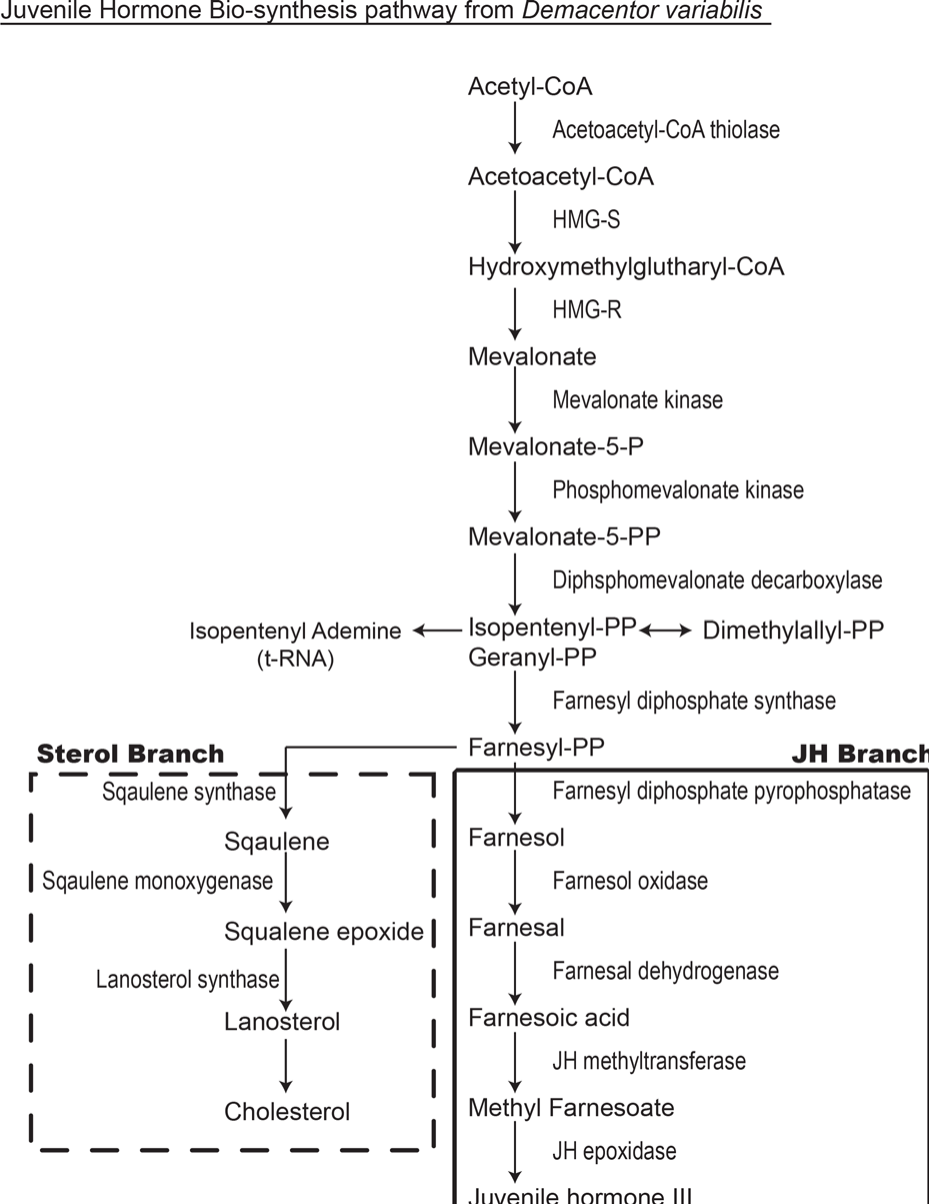
Juvenile hormone III and cholesterol bio-synthesis pathways in insects, modified from Bellés et al. [38].

All of the enzymes that comprise the JH biosynthetic pathway in insects (the mevalonate pathway followed by the JH branch from farnesyl PP to JH III) (Fig 1) are found in the corpora-allata, which is part of the stomatogastric nervous system located ventral and dorsal to the insect brain [41]. The insect brain and ventral nerve cord comprise the central nervous system (CNS) and along with the stomatogastric nervous system regulates neuroendocrine and endocrine functions. Typically, the mevalonate pathway in most animals would lead to the synthesis of steroids, but arthropods, including insects and ticks, have lost the ability to synthesize steroids *de novo* (Fig 1). JH biosynthesis is regulated by neuropeptides allatotropins and allatostatins produced in the brain that regulate JH biosynthesis in the stomatogastric nervous system and more specifically in the corpora allata in insects.

The tick’s synganglion, which is the tissue source for our transcriptomics research, is located in the center of the body in the tick hemocoel and is homologous in insects with (i) the CNS (brain and ventral nerve cord) and (ii) the stomatogastric nervous system which in insects would include the corpora allata, (the site of biosynthesis of JH) [1]. A retrocerebral organ complex is located on the dorsal side of the supraesophageal region of the synganglion in what should be the same region as the insect stomatogastric nervous system. It has also been hypothesized that the lateral segmental organs located in the lateral nerve plexus between the pedal nerves leading from the synganglion may be the site of JH synthesis in ticks, primarily based on the abundance of smooth endoplasmic reticulum in these glands and the histological similarity of these organs to the insect corpora allata [5]. By developing transcriptomes to the entire synganglion including as much of the pedal nerves as possible and the lateral segmental organs, our dissections should include at least the tick equivalent of the insect corpora allata and essentially includes all of the tick CNS and stomatogastric nervous system. The only exceptions for the latter may be the tick equivalent of the insect ingluvial ganglia and nerves leading to the ingluvial ganglia (if present in ticks). For our research, we generated and examined synganglion transcriptomes from three different adult tick species, *D*. *variabilis* and *I*. *scapularis* (hard ticks) and *O*. *turicata* (soft tick). Transcriptomes from two other tissues, *D*. *variabilis* adult midgut and male reproductive system where JH biosynthesis would not be expected based on insect systems, were used for comparisons.

#### *D*. *variabilis* synganglion transcriptome

The transcriptome generated from the adult American dog tick synganglia contained 532,136 filtered and vector-trimmed reads and the average length of the reads was 229 base pairs (bp) (Table 1). Redundant sequences were assembled with the GS Assembler algorithm (Roche Indianapolis, IN, USA) and 21,119 contigs were obtained; the Basic Local Alignment Search Tool or BLAST analysis of the National Center for Biotechnology Information (NCBI) database was conducted to predict putative functions of the contigs. A search against the GenBank nonredundant (nr) database by BLASTx using a translated nucleotide query with an *e*-value cut-off of e-10, resulted in at least one match for 13,344 sequences (63.2% of the expressed genes). There were 6,045 transcripts that had an expected value (*e*-value) of 1e-06 or lower when compared to the GenBank nr database using BLASTx [30].

**Table 1:** Comparison of transcriptomes from different tick species, sexes, tissues and sequencing methods.

Tick	Sequencing method	Sex/Feeding stage/Tissue	Number of reads	Average read length (base pairs)	Number of contigs
***D*. *variabilis***	454 pyrosequencing (GS-FLX)	50 synganglia each from unfed, part-fed virgin and part-fed mated	532,136	229	21,119
***I*. *scapularis***	Illumina Truseq-I	50 synganglia mixed from unfed, part-fed, and replete females	34,520,330	68	41,249
	Illumina Truseq-II	45 part-fed female synganglia	117,900,476	72	30,838
	454 pyrosequencing (GS-FLX)	30 part-fed female synganglia	456,073	267	20,630
***O*. *turicata***	Illumina Truseq	50 replete female synganglia	63,528,102	438	132,258
**^1^*D*. *variabilis***	454 pyrosequencing (GS-FLX)	500 each from male accessory glands, testes vas deferens	563,093	300	12,804
**^2^*D*. *variabilis***	Sanger	^3^unfed and fed ticks of both sexes	23,045	Not Available	1,679

^1^*D*. *variabilis* male reproductive system transcriptome from Sonenshine et al. [35].

^2^*D*. *variabilis* (midgut) transcriptome from Anderson et al. [36].

^3^Number of ticks unknown.

#### *I*. *scapularis* synganglion transcriptome

Three separate transcriptomes were constructed for the black-legged tick (Table 1). For the first Illumina transcriptome (**I**, Table 1) mixed unfed, part-fed and replete female synganglia were extracted to create a cDNA library. Sequencing of this library produced a total of 34,520,330 reads with an average read length of 68 bp. After vector trimming, the raw reads were assembled using CLC-BIO [33] into a total of 41,249 contigs with an average length of 480 bp. BLASTx against the NCBI database was used to predict putative functions of the contigs. Searches against the GenBank nr database using BLASTx were conducted with an *e*-value cut-off of 1e-06. For the second *I*. *scapularis* Illumina transcriptome (**II**, Table 1), part-fed adult female synganglia total RNA was extracted and used to synthesize a cDNA library. After Illumina sequencing, a total of 117,900,476 raw reads were produced with an average length of 72 bp (Table 1). Assembly with the CLC-BIO [33] algorithm produced a total of 30,838 contigs with an average length of 655bp. The putative functions for these contigs were predicted with BLASTx using the NCBI database. The third transcriptome was constructed using 454 pyrosequencing. Part-fed female synganglia total RNA was extracted and a cDNA library constructed followed by 454 pyrosequencing on a GS-FLX sequencer (Roche). Pyrosequencing generated 456,073 total reads with an average length of 267 bp per read (Table 1). The reads were assembled with the CLC-BIO algorithm [33] which produced 20,630 contigs with an average length of 523 bp. BLASTx against the NCBI database was used to predict putative functions of the contigs. Searches for protein function were conducted against the GenBank nr database with BLASTx using a translated nucleotide query, with an *e*-value cut-off of e-10.*O*. *turicata synganlion transcriptome***—**Synganglia were dissected from blood fed female *O*. *turicata*, total RNA extracted and a cDNA library constructed. After Illumina sequencing, a total of 63,528,102 raw reads was generated. The assembly of raw reads was conducted with the CLC-BIO [33] assembly program (CLC BIO, Cambridge, MA) and yielded 132,258 contigs with an average contig length of 438 bp. Batch BLAST from Blast2go pro [42] was used to compare these contigs with the nr database from NCBI to establish putative functions with an e-value cut off of e-6.

#### Additional comparative transcriptomes

Our research results with synganglion transcriptomes were compared with transcriptomes from two additional tissues exclusive of the CNS, namely, the male reproductive system and midgut of the American dog tick. The former was constructed using 500 part fed adult males accessory glands, testes and vas deferens and 454 pyrosequencing produced 563,093 reads which averaged 300 bp per read and assembled into 12,804 contigs (Table 1). Putative functions were determined by BLASTx against the NCBI database. A total of 3,951 contigs were found to match known genes, with an e-value cut-off of e-10, when compared to the GenBank nr database by BLASTx [35]. The midgut transcriptome was developed from unfed and fed male and female adults and was conducted by our group prior to the availability of 454 or Illumina sequencing. We obtained 23,045 reads which were assembled using the CAP2 assembler [43] into 1,679 contigs (Table 1) [36]. The contigs were initially identified with BLASTx, BLASTn, and ROS-BLAST using the nr NCBI protein database. Then, the characterized proteins were compared to a custom-prepared ACARI database, the Gene Ontology (GO) database, and to the NCBI conserved domains database (CDD) including KOG, PFAM and SMART for putative functional assignments.

### Putative mevalonate (farnesyl-PP) pathway in the tick synganglion

The initial steps in JH biosynthesis comprise the mevalonate or farnesyl-PP pathway (Fig 1). This pathway is important in many organisms leading to the steroid (cholesterol) biosynthesis pathway. Steroids are essential in membrane function and comprise the structure of hormones essential for life. Insects do not synthesize cholesterol de novo [38,44], and we unable to find squalene synthase, squalene monoxygenase and lanosterol synthase involved in steroid synthesis (Fig 1) in any of our transcriptomes. The mevalonate pathway uses acetyl-CoA as a starting material which undergoes condensation with another acetyl-CoA subunit via acetoacetyl-CoA thiolase to form acetoacetyl-CoA. Then acetyl-CoA condenses with acetoacetyl-CoA to form 2-hydroxy-3-methylglutaryl-CoA (HMG-CoA) through HMG-CoA synthase (HMG-S). HMG-CoA then is reduced to mevalonate by NADPH with the enzyme HMG-CoA reductase (HMG-R) (Fig 1), which is the rate limiting step in cholesterol synthesis. HMG-R is an important regulator step in steroid biosynthesis and has been the focus of research into the development of anti-cholesterol drugs. Mevalonate is phosphorylated by mevalonate kinase (MK) to 5-phosphomevalonate, also known as phosphomevalonic acid. Then the enzyme phosphomevalonate kinase phosphorylates 5-phosphomevalonate to 5-pyrophosphomevalonate (mevalonate-5-PP). The enzyme, diphosphomevalonate decarboxylase metabolizes mevalonate-5-PP to isopentenyl diphosphate (IPP) (Fig 1). The C-5 isoprene unit is used by prenyltransferases to build prenyl chains whose carbon atom numbers are typically in multiples of five. Biogenesis of the sesquiterpene precursor of JH III, farnesyl diphosphate (FPP), is achieved by FPP synthase (FPPS, a prenyltransferase) which catalyzes the head to tail condensation of three isoprene units. In the latter reaction, the chain initiator is the allylic isomer of IPP, dimethyl allyl diphosphate (DMAPP), the production of which is catalyzed by an IPP isomerase (IPPI) [37, 38, 41, 45].

In the past, identification of precursors of JH or JH itself in ticks were conducted by the chemical isolation of the products in the mevalonate-JH biosynthetic pathway [22, 24, 46]. This work was challenging in insects because of the small size of the corpora allata, the source of JH, the difficulty of obtaining a clean dissection of the corpora allata, and the extremely low production levels of the hormones. Tracer studies using radiolabeled acetate have also been used in insects and ticks [22]; this approach is problematic because of variations in enzymatic rates at each step of biosynthesis (which makes detection of intermediates difficult), organ culture methods are needed that mimic *in vivo* conditions and again, there is the need to dissect the corpora allata. These problems are even more challenging for the study of ticks because (i) their small size compared to many insects, (ii) ticks do not have a distinct head region, (iii) the stomatogastric and CNS are condensed into a single synganglion, and (iv) tick rearing itself is difficult requiring feeding on a live animal host at each stage of its development. For these reasons and as an alternative approach, we conducted in this study the first global analysis of gene expression in ticks for the enzymes in the mevalonate-JH III biosynthesis pathway examining the synganglion of three tick species representing hard and soft ticks; the goal was to determine whether adult ticks have the potential of making JH III or its precursors by the detection of the enzymes that comprise the JH III biosynthesis pathway (Fig 1). Our focus was JH III biosynthesis since this pathway has been well characterized in insects [38] including our recent identification of the entire pathway in the genome of the termite [47]. Also, the synthesis of JH III is exclusive to the lower insects and therefore most likely to also be found in ticks.

**Fig 2:**
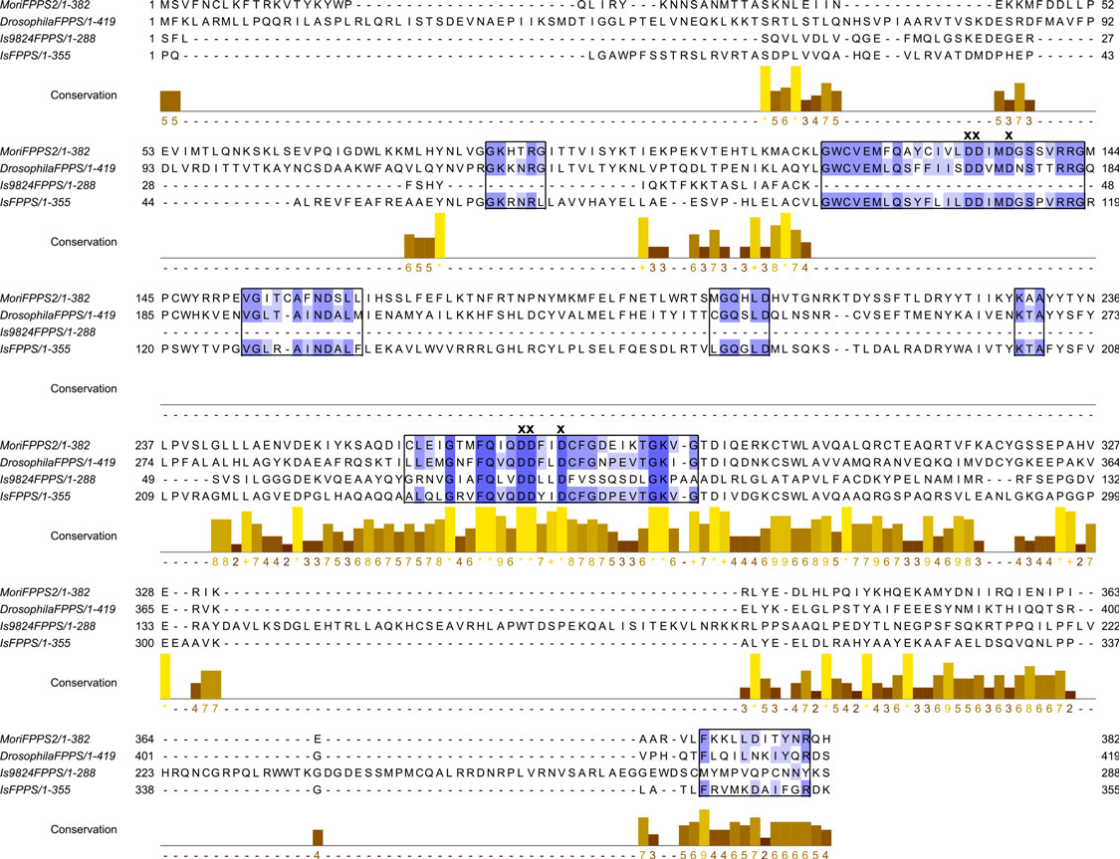
Sequences alignment of farnesyl diphosphate synthase (FPPS2) from *Bombyx mori* and *Drosophila melanogaster* with contig 9824 and XP_002408650 from I. scapularis transcriptome and genome. Farnesyl diphosphate synthase (FPPS2) described from *Bombyx mori* and *Drosophila melanogaster* aligned with contig 9824 from the *I*. *scapularis* transcriptome and FPPS (XP_002408650) from the *I*. *scapularis* genome. The boxed regions are the seven prenyltransferase conserved regions previously identified by Koyoma et al. [50]. “X” above the amino acids indicates position of Asp residues within the two aspartate-rich domains. Below the sequence alignment is the conservation panel which is measured as a numerical index (9–0) reflecting the conservation of physicochemical properties in the alignment. * (asterisk) denotes the highest identity score (identical residues in all species) followed by a score of 9 for the next most conserved group of residues containing substitutions by amino acids included in the same physicochemical class as described by Livingstone and Barton [52].

A BLASTx and BLASTn search of the synganglion transcriptomes of *D*. *variabilis*, *I*. *scapularis* and *O*. *turicata* (Table 1) for the insect enzymes involved in the synthesis of JH III (Fig 1) was successful in identifying all of the transcripts of the enzymes involved in farnesyl-PP biosynthesis (Table 2). The putative messages characterized were acetoacetyl-CoA thiolase (60% identical to insects), hydroxymethylglutaryl-CoA synthase (60% identical to insects), hydroxymethylglutaryl-CoA reductase (50% identical to insects), mevalonate kinase (38% identical to insects), phosphomevalonate kinase (52% identical to insects), diphosphomevalonate decarboxylase (54% identical to insects), and farnesyl diphosphate synthase (64% identical to insects) among the three transcriptomes examined. The top three insect BLAST matches for *D*. *variabilis*, *I*. *scapularis* and *O*. *turicata* in the mevalonate pathway are listed with contig number, length (bp), e-value (ranging from 0.0 to 6.3), and percent identity (Table 2). For *O*. *turicata*, all of the enzymes in the mevalonate pathway was found except farnesyl diphosphate synthase (the last step for the synthesis of farnesyl-PP); however, this enzyme was found in both the *D*. *variabilis* and *I*. *scapularis* transcriptomes with the lowest e-values in the e-11 and e-12 range, respectively. Matches for many of the other enzymes in the mevalonate pathway were found in *D*. *variabilis* and *I*. *scapularis* with the best matches occurring in the forming ranging from e-14 to e-42 (Table 2). A BLASTx and BLASTn search of the *I*. *scapularis* genome for the insect enzymes involved in the synthesis of juvenile hormone (JH) III [48] revealed the presence of all but two of the enzymes involved in the farnesyl-PP pathway. The genes found were acetoacetyl-CoA thiolase, hydroxymethylglutaryl-CoA synthase, hydroxymethylglutaryl-CoA reductase, mevalonate kinase, phosphomevalonate kinase, diphosphomevalonate decarboxylase and farnesyl diphosphate synthase. The top insect BLAST results from these *I*. *scapularis* messages had *e*-values ranging from *e*-44 to 0.0. Isopentenyl diphosphate isomerase and geranyl diphosphate synthase were not found. Fig 2 shows the farnesyl diphosphate synthase (FPPS) contig 9824 (KT728823) from the *I*. *scapularis* transcriptome and FPPS (XP_002408650) from the *I*. *scapularis* genome (www.vectorbase.org) aligned with FPPS described in *Bombyx mori* and *Drosophila melanogaster* [49]. In the alignments, seven prenyltransferase conserved regions previously identified by Koyama et al. [50] are highlighted in boxes. Only the last two conserved regions were found for contig 9824 because this contig from the *I*. *scapularis* transcriptome is not a full length sequence. But all seven prenyltransferase conserved regions were found in the FPPS sequence from the *I*. *scapularis* genome; also the region II and VI two aspartate-rich motifs (marked with “X” on top) were identified (Fig 2), further evidence for the presence of FPPS in the synganglion and genome of *I*. *scapularis*. The FPPS sequence from the *I*. *scapularis* genome shares 43% identity with the FPPS found in *B*. *mori* (e-value of e-85); the FPPS (contig 9824) from the *I*. *scapularis* transcriptome shared 27% identity with an e-value of 6e-05. Even with 27% sequence identify, both messages share a high level of amino acid conservation. FPPSs are unique and diversified. There are three different copies of FPPS in *B*. *mori*, six in the honey bee, *Apis mellifera*, two in the monarch butterfly, *Danaus plexippus*, and one each in the fruit fly, *Drosophila melanogaster*, the malaria mosquito, *Anopheles gambiae* and red flour beetle, *Tribolium castaneum* [51]. Two FPPS messages were found between the *I*. *scapularis* synganglion transcriptome and genome (Fig 2).

**Table 2:** Longest contig from the synganglion transcriptomes of D. variabilis, I. scapularis, and O. turicata with the lowest e-values for matches in the mevalonate pathway (leading to the synthesis of juvenile hormone III) in insects^1^.

	*Dermacentor variabilis*			*Ixodes scapularis*			*Ornithodoros turicata*		
Substrate	Contig#/Accession#	Top 3 insect matches	e-value	Contig#/Accession#	Top 3 insect matches	e-value	Contig #/Accession #	Top 3 insect matches	e-value
**Enzyme**	(length, bp)	(Accession #)	(% identity)	(length, bp)	(Accession #)	(% identity)	(length, bp)	(Accession #)	(% identity)
Acetyl-CoA	19096/KT602361	*M*. *hirsutus*	4e-22	11699/KT728819	*A*. *darlingi*	8e-93	74522/KT602365	*A*. *aegypti*	5e-122
**Acetoacetyl-CoA Thiolase**	(298)	ABN11931.1	(61%)	(222)	ADMH01001403.1	(56%)	(773)	XP_001657918.1	(65%)
		*N*. *vitripennis*	2e-21		*A*. *aegypti*	2e-92		*D*. *simulans*	5e-119
		XP_001606375.1	(60%)		XM_001654701.1	(57%)		XP_002076898.1	(66%)
		*A*. *pisum*	3e-21		*A*. *aegypti*	4e-92		*D*. *mojavensis*	6e-119
		XP_001943983.1	(61%)		DQ440481.1	(57%)		XP_002009553.1	(65%)
Acetoacetyl-CoA	11805/KT602360	*A*. *gambiae*	1e-13	NA^2^	NA	NA	5140/KT602366	*G*. *morsitans*	0.0
**HMG-S**	(231)	XP_315872.3	(57%)				(2758)	ADD19375.1	(62%)
		*D*. *jeffreyi*	2e-13					*B*. *germanica*	0.0
		AAF89580.1	(50%)					P54961.1	(62%)
		*B*. *germanica*	2e-13					*N*. *vitripennis*	0.0
		P54961.1	(59%)					XP_003426942.1	(62%)
HMG-CoA	4404/KT602359	*I*. *paraconfusus*	5e-42	NA	NA	NA	112681/KT602367	*G*. *viridula*	3e-114
**HMG-R**	(525)	AAD20975.2	(77%)				(1851)	ABO37161.1	(45%)
		*C*. *floridanus*	1e-41					*P*. *cochleariae*	4e-114
		EFN61977.1	(70%)					ABO37160.1	(45%)
		*N*. *vitripennis*	2e-41					*B*. *impatiens*	7e-114
		XP_001601404.1	(70%)					XP_003492098.1	(43%)
Mevalonate	NA	NA	NA	NA	NA	NA	67236/KT602369	*B*. *impatiens*	7e-04
**Mevalonate Kinase**							(313)	XP_003492382.1	(38%)
								*D*. *simulans*	9e-04
								XP_002107342.1	(37%)
								*A*. *gambiae*	9e-04
								XP_313644.5	(48%)
Mevalonate-5-P	3143/KT602372	*B*. *mori*	5e-14	11919/KT728820	*A*. *cephalotes*	9e-12	42225/KT602369	*S*. *invicta*	4e-52
**Phosphomevalonate Kinase**	(330)	BAF62110.1	(46%)	(333)	XP_012063437.1	(60%)	(1020)	EFZ11390.1	(50%)
		*H*. *saltator*	2e-13		*P*. *barbatus*	6e-11		*A*. *echinatior*	5e-50
		EFN87008.1	(44%)		XP_011641710.1	(60%)		EGI69273.1	(47%)
		*C*. *floridanus*	2e-12		*S*. *invicta*	7e-11		*A*. *florea*	9e-50
		EFN64677.1	(44%)		XP_011170107.1	(52%)		XP_003695278.1	(48%)
Mevalonate-5-PP	NA	NA	NA	9701/KT728825	*D*. *mojavensis*	2.8	5162/KT602370	*D*. *ponderosae*	5e-141
**DiphosphomevalonateDecarboxylase**				(208)	XP_002003397.1	(56%)	(1544)	AFI45055.1	52%
					*A*. *darling*	6.3		*A*. *mellifera*	2e-140
					EFR19559.1	(34%)		XP_001121619.2	51%
					*T*. *castaneum*	6.3		*A*. *aegypti*	3e-139
					EEZ98110.0	(37%)		XP_001648384.1	23%
Isopentenyl-PP–Geranyl-PP	4375/KT602363	*D*. *jeffreyi*	7e-11	10790/KT728821	*A*. *darling*	5e-12	NA	NA	NA
**Farnesyl Diphosphate Synthase**	(241)	AAX78435.1	(57%)	(461)	EFR25052.1	(71%)			
		*D*. *yakuba*	3e-10		*A*. *gambiae*	5e-12			
		XP_002090289.1	(62%)		XP_316306.3	(71%)			
		*D*. *sechellia*	3e-10		*S*. *invicta*	7e-12			
		XP_002033374.1	(62%)		EFZ14302.1	(60%)			

^1^ *Acromyrmex echinatior*, leaf-cutter ant; *Acyrthosiphon pisum*, pea aphid; *Aedes aegypti*, yellow fever mosquito; *Anopheles darlingi*, American malaria mosquito; *Anopheles gambiae*, African malaria mosquito; *Apis florea*, red dwarf honey bee; *Apis mellifera*, western honey bee; *Atta cephalotes*, leaf-cutter ant; *Blattella germanica*, German cockroach; *Bombus impatiens*, eastern bumble bee; *Bombyx mori*, silkworm; *Camponotus floridanus*, carpenter ant; *Dendroctonus ponderosae*, mountain pine beetle; *Drosophila mojavensis*, fruit fly; *Drosophila simulans*, fruit fly; *Drosophila yakuba*, fruit fly; *Gastrophysa viridula*, green dock beetle; *Glossina morsitans*, Savannah tsetse fly; *Harpegnathos saltator*, Indian jumping ant; *Ips* paraconfusus, bark beetle; *Maconellicoccus hirsutus*, mealybug; *Nasonia vitripennis*, jewel wasp; *Phaedon cochleariae*, mustard leaf beetle; *Pogonomyrmex barbatus*, red harvester ant; *Solenopsis invicta*, red imported fire ant; *Tribolium castaneum*, red flour beetle.

^2^NA (not available), no match found.

The mevalonate pathway is an important metabolic pathway that exists in all higher eukaryotes and many bacteria and is involved in multiple metabolic functions. For example, it can play a role in cholesterol synthesis and maintaining membrane structure. The pathway is also involved in membrane-bound protein prenylation, the addition of hydrophobic molecules to proteins, and important in cell signaling and carcinogenesis. The mevalonate pathway contributes to the synthesis of heme A and ubiquinone that participate in electron transport across cellular membranes and the synthesis of isopentenyl adenine, which is present in some transfer RNA. The pathway can also produce hormonal messengers such as cytokinins and phytoalexins in plants, steroid hormones in mammals [53], defensive secretions, pheromones, and JH in insects [54], [38]. Since its final product is typically cholesterol that functions in maintaining cell membranes, most of the research on the mevalonate pathway has focused on vertebrate systems and its close association with human cardiovascular diseases [55].

Insects cannot synthesize cholesterol since they lack squalene synthase, squalene monooxygenase and lanosterol synthase in the sterol branch (Fig 1). We have identified all of the enzymes involved in the mevalonate pathway from acetyl-CoA to FPP in the three tick transcriptomes that were examined (Table 2) and part of the pathway in the *I*. *scapularis* genome [48]. The mevalonate pathway was also found in the spider mite, *Tetranychus urticae*, genome, a close relative to ticks [56]. Although the mevalonate pathway has other functions such as ubiquinone synthesis, dolichol synthesis and protein prenylation [44], we were not able to find any of the enzymes that make up this pathway in the *D*. *variabilis* male reproductive or midgut transcriptomes examined. Because of the ubiquitous distribution of fat body in ticks and its deposition on trachea, it is unlikely that fat body messages were not present in any of the transcriptomes that were examined. The evidence so far suggests that the mevalonate (farnesyl-PP) pathway is present only in the synganglion which is inclusive of the stomatogastric nervous system and the likely tick equivalent of the insect corpora allata. Its presence only in the synganglion further suggests that, like in insects, the pathway is involved in a regulatory function and possibly is contributing to the production of a JH precursor beyond farnesyl-PP (further discussion follows).

### Putative farnesal branch in ticks

Following the mevalonate pathway, there are two metabolic routes possible: the steroid biosynthetic pathway and the JH branch (Fig 1). In most animals, the final product of the mevalonate pathway is the synthesis of steroids like cholesterol. Insects lack the genes required for the production of cholesterol from farnesyl-PP; this include squalene synthase which catalyzes the reductive condensation of farnesyl-PP [44]; [57]; [38]. Searching through our tick transcriptomes (Table 1), we were not able to find any of the enzymes that make up the steroid biosynthesis pathway.

The JH branch (farnesyl-PP to JH III) found in insects involves five enzymes: farnesyl diphosphate pyrophosphatase, farnesol oxidase, farnesal dehydrogenase, JH methyl transferase and JH epoxidase (Fig 1). A BLASTx and BLASTn search of our synganglion transcriptomes of *D*. *variabilis*, *I*. *scapularis* and *O*. *turicata* (Table 1) for the insect enzymes involved in the JH branch (Fig 1) was successful in identifying (i) farnesol oxidase, a short chain dehydrogenase implicated in the conversion of farnesol to farnesal, and (ii) a methyltransferase that potentially could add a methyl ester to farnesoic acid or JH acid to produce methyl farnesoate or JH III, respectively (Table 2). We did not search for farnesyl diphosphate pyrophosphatase and farnesal dehydrogenase in the JH branch since these enzymes have not been identified yet in insects [38].

#### Absence of a JH epoxidase (CYP15A1) in ticks

**Fig 3:**
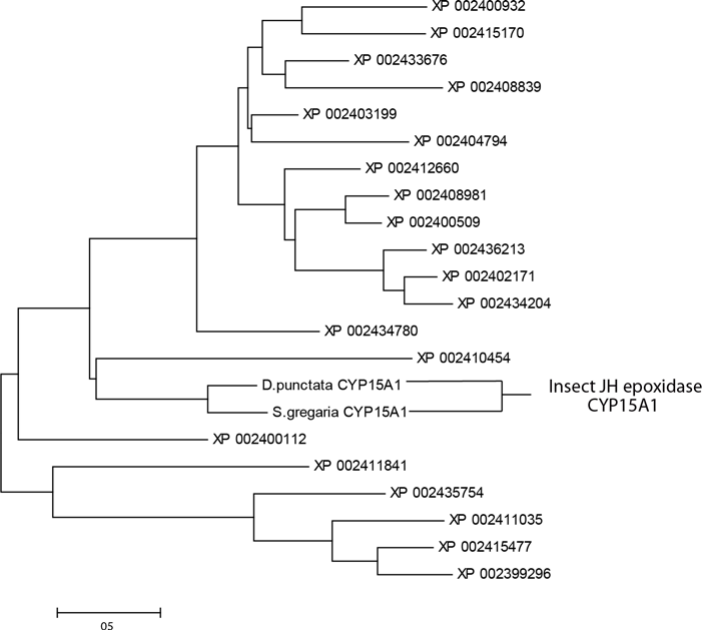
Phylogenetic tree comparing the P450 CYP15A1 found in *D*. *punctata* and *S*. *gregaria* to P450s found in *I*. *scapularis* genome. Phylogenetic tree comparing the P450s CYP15A1 known in *D*. *punctata* (AAS13464) and *S*. *gregaria* (HQ634703) to add the C10,11 epoxide to methyl farnesoate to make JH III, to the P450s in the *I*. *scapularis* genome with the top BLASTp matches (lowest e-values) to the *D*. *punctata* CYP15A1 and which had the longest length in base pairs. The 20 full length CYP messages with the lowest e-values and maximum length available from *I*. *scapularis* were not in the CYP15A1 family (in a different clade) as shown. The optimal neighbor-joining phylogenic tree was constructed by the Molecular Evolutionary Genetics Analysis (MEGA) program. All of the accession numbers labeled with XPs are CYP messages from the *I*. *scapularis* genome. The recombinant expressed CYP15A1s from *D*. *punctata* and *S*. *gregaria* are labeled insect JH epoxidase. Percent identity was determined by BLASTp. The highest identity message XP_002410454 shares 28% identity with *D*. *punctata* and 30% identity with *S*. *gregaria*. No members of the Cyp15A family were found in the *I*. *scapularis* genome [48]. An alignment of the closest sequence XP002410454 to the CYP15A1 family is shown in Fig 4.

**Fig 4:**
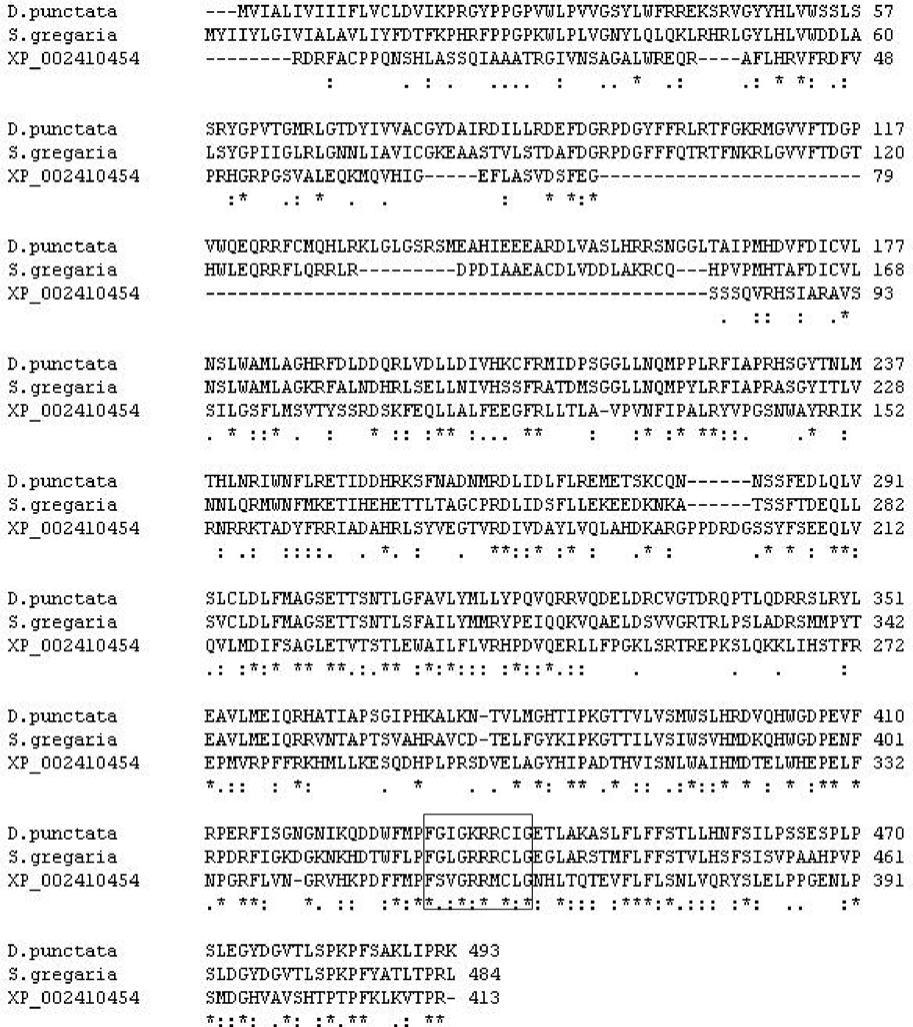
Sequence alignment of XP_002410454 from the *I*. *scapularis* genome with CYP15A1 from *D*. *punctata* AAS13464 and *S*. *gregaria* HQ634703. Sequence alignment (pairwise) for XP_002410454, the top BLAST match from the *I*. *scapularis* genome to that of the published sequences (GenBank) for CYP15A1 from *D*. *punctata* AAS13464 [23] and *S*. *gregaria* HQ634703 [59]. The JH epoxidase is a member of the CYP15A1 family and has been cloned and characterized from the corpora allata of *D*. *punctata* and *S*. *gregaria*. It has been demonstrated this enzyme can add an epoxide to the C10,11 position of methyl farnesoate to produce JH III with high stereo selectivity (10R). All members of the family CYP15 share >40% identity at the amino acid level and >55% identity at the subfamily level [58]. The boxed sequence on the alignment is the signature heme-binding motif of the P450 (F**G***C*G). An * (asterisk) indicates positions which have a single, fully conserved residue. A colon indicates conservation between groups of strongly similar properties, scoring > 0.5 in the Gonnet PAM 250 matrix. A period indicates conservation between groups of weakly similar properties, scoring ≤ 0.5 in the Gonnet PAM 250 matrix. Based on our alignment, the *I*. *scapularis* sequence XP_002410454 only shares 28% identity with *D*. *punctata* and 30% identity with *S*. *gregaria*. Therefore XP_002410454 is not a member of the CYP15 gene family.

The last step in JH biosynthesis in insects is the epoxidation of methyl farnesoate or farnesoic acid by a P450 which adds a C10,11 epoxide with a specific *R*-enantiomeric C10 asymmetric carbon to produce JH III or JH III acid, respectively. The JH epoxidases so far characterized in insects are in the P450 family CYP15A1. An absolute functional assignment has been demonstrated in insects to this P450 family, where the CYP15A1 message was cloned from the corpora allata of the cockroach, *Diploptera punctata*, and shown to add an epoxide at the proper location with C-10*R* high stereo selectivity [23]. All members of the CYP15 family share >40% identity at the amino acid level and those with >55% identity are in the CYP15A subfamily [58]. We were not able to find any JH epoxidases in the CYP15A1 family in any of our tick transcriptomes (Table 3) or in the *I*. *scapularis* genome [48]. We found two hundred P450 transcripts in the *I*. *scapularis* genome that varied in length (not all were complete transcripts), but none were specific to the CYP15A1 subfamily. Fig 3 shows the alignment of XP_002410454 from the *I*. *scapularis* genome with CYP15A1 from *D*. *punctata* and *S*. *gregaris* (the latter also shown to add an epoxide to make JH III; [23]). XP_002410454 is a full-length sequence we identified in the *I*. *scapularis* genome that shared the highest identity and lowest e-value with the JH epoxidase CYP15A1 in *D*. *punctata*. The signature heme-binding region (F**G***C*G) was found in all of the P450s aligned as expected including XP_002410454, suggesting the latter is a P450. A phylogenetic tree was constructed for the top 20 (based on the lowest e-values compared to the cockroach JH epoxidase), full-length sequences in the *I*. *scapularis* genome to CYP15A1 from *D*. *punctata* and *S*. *gregaris* (Fig 4). The *D*. *punctata* and *S*. *gregaris* enzymes are in the same clade as would be expected. XP_002410454 also had the closest relationship with CYP15A1 but shared only 28% identity with *D*. *punctata* JH epoxidase placing it outside of the CYP15A1 family and subfamily. CYP15A1 also was not found in the spider mite genome [56].

**Table 3:** Longest contig from the synganglion transcriptomes of D. variabilis, I. scapularis, and O. turicata with the lowest e-values for matches in the JH branch in insects^1^.

	*Dermacentor variabilis*			*Ixodes scapularis*			*Ornithodoros turicata*		
Substrate	Contig#/Accession#	Top 3 insect matches^2^	e-value	Contig#/Accession#	Top 3 insect matches^2^	e-value	Contig #/Accession #	Top 3 insect matches^2^	e-value
Enzyme	(length, bp)	(Accession #)	(% identity)	(length, bp)	(Accession #)	(% identity)	(length, bp)	(Accession #)	(% identity)
Farnesyl-PP	NA^3^	NA	NA	NA	NA	NA	NA	NA	NA
**Farnesyl Diphosphate Pyrophosphatase**									
Farnesol	5964/KT02362	*N*. *vitripennis*	1e-102	8342/KT728824	*C*. *floridanus*	1e-102	NA	NA	NA
**Farnesol Oxidase**	(758)	XP_001606362.2	(53%)	(440)	EFN72306.1	(58%)			
		*D*. *ananassae*	5e-101		*S*. *invicta*	5e-101			
		XP_001960084.1	(54%)		EFZ18513.1	(54%)			
		*D*. *willistoni*	1e-99		*C*. *capitata*	1e-99			
		XP_002068852.1	(56%)		XP_004523325.1	(56%)			
		***A*. *aegypti***^*4*^	**3e-12**		***A*. *aegypti***	**8e-21**			
		**D2WKD9**	**(25%)**		**D2WKD9**	**(32%)**			
Farnesal	NA	NA	NA	NA	NA	NA	NA	NA	NA
**Farnesal Dehydrogenase**									
Farnesoic acid	19251/KT602364	***B*. *mori***	**4e-25**	24500/KT728822	*A*. *darlingi*	4e-24	16407/KT602371	***A*. *aegypti***	**4e-34**
**Juvenile Hormone Methyltransferase**	(1014)	**NP_001036901**	**(30%)**	(649)	ETN59754.1	(39%)	(757)	**XP_001651876.1**	**(38%)**
		***T*. *castaneum***	**2e-24**		***T*. *castaneum***	**2e-24**		*S*. *litura*	1e-32
		**NP001120783**	**(33%)**		**NP001120783**	**(32%)**		BAF63629.1	(37%)
		***A*. *aegypti***	**1e-18**		***D*. *melanogaster***	**9e-24**		***B*. *mori***	**5e-30**
		**XP_001651876.1**	**(32%)**		**NP_609793**	**(35%)**		**NP_001036901**	**(35%)**
		*A*. *pisum*	1e-18		*S*. *gregaria*	5e-23		*H*. *armigera*	1e-28
		NP_001156251.1	(35%)		ADV17350.1	(39%)		BAF63630.1	(35%)
		***D*. *melanogaster***	**2e-18**		***A*. *aegypti***	**8e-23**		***D*. *melanogaster***	**4e-26**
		**NP_609793**	**(25%)**		**XP_001651876.1**	**(32%)**		**NP_609793**	**(34%)**
		*C*. *floridanus*	2E-17		***B*. *mori***	**1e-19**		***T*. *castaneum***	**2e-25**
		EFN68634.1	(28%)		**NP_001036901**	**(31%)**		**NP001120783**	**(38%)**
Methyl farnesoate	NA	NA	NA	NA	NA	NA	NA	NA	NA
**JH epoxidase**									
Juvenile hormone III									

^1^ *Acyrthosiphon pisum*, pea aphid; *Aedes aegypti*, yellow fever mosquito; *Anopheles darlingi*, American malaria mosquito; *Bombyx mori*, silkworm; *Camponotus floridanus*, carpenter ant; *Ceratitis capitata*, Mediterranean fruit fly; *Drosophila ananassae*, fruit fly; *Drosophila melanogaster*, fruit fly; *Drosophila willistoni*, fruit fly; *Nasonia vitripennis*, jewel wasp; *Schistocerca gregaria*, desert locust; *Solenopsis invicta*, red imported fire ant; *Spodoptera litura*, leafworm/Noctuid moth; *Tribolium castaneum*, red flour beetle.

^2^The order of BLAST results are listed based on e-value.

^3^NA (not avaiable), no match found.

Neese et al. [22] was unable to find that ticks could synthesize JH either *in vivo* or *in vitro* using different tissues, including the synganglion, using a highly sensitive radiotracer method in hard and soft ticks. In addition, these authors could not find any of the insect JHs in the hemolymph of vitellogenic hard and soft ticks by EI GC-MS nor could they find JH in hard ticks using the insect *Galleria* bioassay for JH. Furthermore, there is no consistent evidence that JH or JH mimics when topically applied affect tick development or will induce vitellogenesis [1]. The lack of CYP15A1 in multiple synganglion transcriptomes from hard and soft ticks (Table 3), in the genome of *I*. *scapularis* [48], and in the genome of the spider mite [56], further supports the hypothesis that ticks do not make JH III or use JH in the regulation of their development.

#### JH acid methyltransferase (JHAMT) in ticks

The enzymes involved in the last two steps in JH biosynthesis in insects, JH acid methyltransferase (JHAMT) and JH epoxidase (Fig 1), have been successfully validated at the functional level through cloning, expression and substrate metabolism for their role in the synthesis of JH III [60]; [61]; [62]; [63]. The order of metabolism appears to be variable depending on the insect; in the Lepidoptera, epoxidation precedes esterification by JHAMT [64] and in the Orthoptera, Dictyoptera, Coleoptera and Diptera the reverse occurs [65–69]. JHAMT transfers a methyl group from S-adenosyl-L-methionine (SAM) to farnesoic acid or JH acid depending on the insect. Thus, JHAMTs must have a conserved SAM binding motif which is typical of the general SAM-dependent methyltransferase family [38].

**Fig 5:**
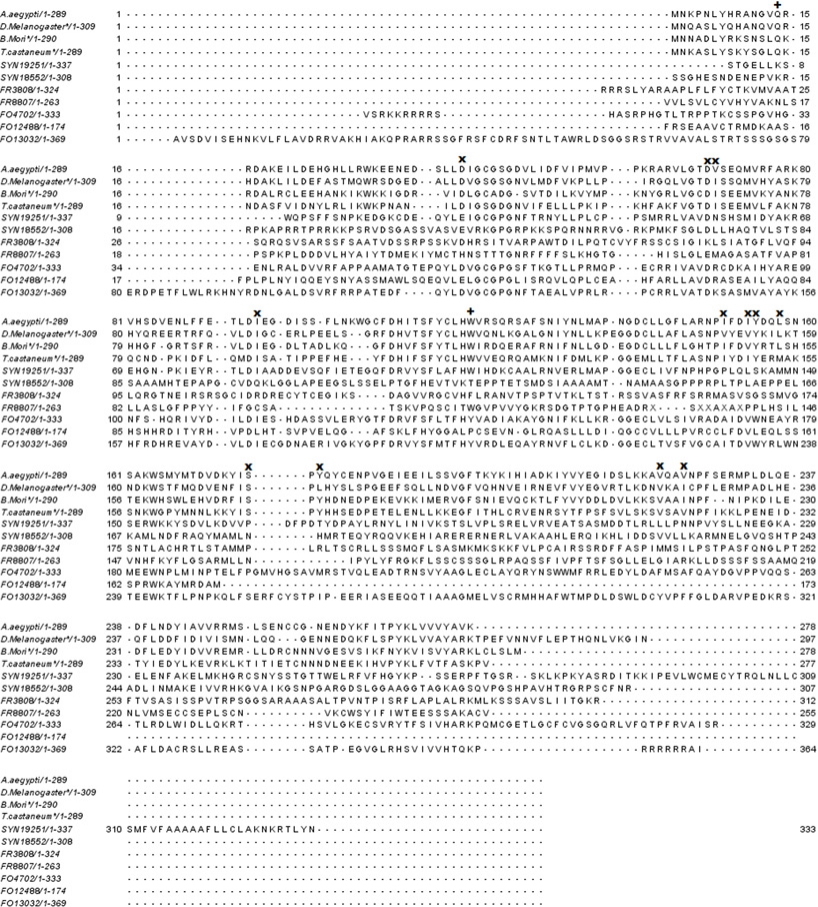
Alignment of methyltransferases from ticks with that of known JH methyltransferases from insects (based on published direct demonstration of function or by bioinformatics). Contains the Gln-14 and Trp-120 residues which are important for farnesoic acid or JH acid interaction, and part of the SAM binding motif. Both Gln-14 and Trp-120 residues are labeled with **+** on the top of the sequence. The SAM binding motif and ligand interactions are marked with **X** on the top of the sequence. Selected methyltransferases with the maximum length in bps from the transcriptomes of *D*. *variabilis*, *I*. *scapularis* and *O*. *trunicata* were aligned with known insect JH methyl transferases from *Bombyx mori* (NP_001036901), *Drosophila melanogaster* (NP_609793), *Tribolium castaneum* (NP_001120783), and *Aedes aegypti* (XP_001651876).

JHAMTs have been found and characterized in insects but not in ticks. We found over 100 different methyltransferases in each of our tick synganglion transcriptomes, and there is evidence from the study of the *I*. *scapularis* genome of a large expansion of this gene family [48]. The expansion of this gene family is not found in any insect so far studied. The methyltransferases with the top two highest matches to putative insect acid methyltransferases based on e-values are shown in Table 3 along with the matches to functionally proven JHAMTs (shown in bold). For the latter for *D*. *variabilis*, the e-values range from e-25 to e-18, for *I*. *scapularis* e-24 to e-19 and for *O*. *turicata* from e-34 to e-25 (Table 3). These data alone suggest that ticks have a JHAMT in their synganglia. Methylases including methyltransferases are responsible for transferring a methyl group from a donor to an acceptor substrate, and there are many types of methyltransferases in insects, e.g., DNA methyltransferase, histone methyltranferase, JHAMT and others. The putative association of tick methyltransferases as JHAMTs in Table 3 based on low e-values and high identity has to be considered with caution, since most methyltransferases identified in insects have been associated with JH biosynthesis. Also note in Table 3, there were matches ranging from e-32 to e-17 for acid methyltransferases which have not been functionally proven to be involved in JH synthesis. Selected methyltransferases of maximum length from the transcriptomes of *D*. *variabilis*, *I*. *scapularis* and *O*. *trunicata* were further aligned with insect acid transferases that are reported in the literature to be JHAMTs from *Bombyx mori* (NP_001036901), *Drosophila melanogaster* (NP_609793), *Tribolium castaneum* (NP_001120783), and *Aedes aegypti* (XP_001651876). Recombinant JHAMTs from *Bombyx mori* [60], *Drosophila melanogaster* [61], *Tribolium castaneum* [62], and *Aedes aegypti* [63] were shown to convert farnesoic acid into methyl farnesoate, as well as JHA into JH III and therefore are functionally proven JHAMTS which have the ability to metabolize both farnesoic acid and JHA [70]. The substrate (farnesoic acid or JH acid)-binding site is highlighted with "+" in Fig 5, along with the residues (labeled with X) that interact with the methyl donor (S-adenosylmethionine) and the carboxylic acid of the substrate methyl acceptors, farnesoic acid or JH acid. Interestingly, most of the amino acids important in interactions with the methyl donor (SAM) and the substrate (farnesoic acid or JHA) are highly conserved in the insect JHAMT sequences. The insect JHAMTs and the methyltransferases from the *D*. *variabilis* transcriptome have the classical SAM binding motifs, suggesting they are all methyltransferases. Two critical (conserved) residues near SAM, Gln-14(Q) and Trp-120 (W), bind the carboxyl group of farnesoic acid or JHA and place them in a suitable conformation for catalysis. In alignment (Fig 5), those two sites were labeled with a "+". Both syn19251 and syn18552 from the *D*. *variabilis* transcriptome lack the Gln-14(Q) and Trp-120 (W). Instead they have Lys (K) and Phe (F) in the place of Gln (Q). Amino acid Gln has a polar uncharged (neutral) side chain, but Lys has a positively charged side chain and Phe (F) has a hydrophobic ring on its side chain. Since they have different physical and chemical properties compare to Gln-14, they are structurally not conserved compared with the insect JHAMTs. Overall, the tick acid methyltransferases do not share the same key motifs as for the insect JHAMTs in the substrate binding site. We can confirm the presence of the key SAM-binding motifs in almost every tick methyltransferase we examined (Fig 5) but we are unable to locate a complete substrate-binding site for the JH precursors in the tick acid methyltransferases.

Neese et al. [22] incubated synganglion from the hard tick, *D*. *variabilis*, and the soft tick, *Onithodoros parkeri* in organ culture with a high specific activity, ^3^H-methyl methionine; no ^3^H-methyl JH or ^3^H-methyl farnesoate could be found in the synganglion or culture media. These studies also were conducted with and without the addition of farnesoic acid. In both cases, radiolabeled methyl farnesoate and JH were not found while the same experimental conditions were successful in producing these products in a positive, insect corpora allata control. Furthermore, Neese et al. [22] were unable to find methyl farnesoate as well as the insect JHs in the hemolymph of vitellogenic ticks of both of these same tick species by EI GC-MS. Interestingly, methyltransferases were also found in the spider mite genome and these methyltransferases also lacked the insect substrate binding residues like in ticks (data analysis not shown), but methyl farnesoate was reported by GC-MS in the spider mite. Acid methyl transferases were not exclusive to the synganglion in ticks but were also found in the male reproductive organs and midgut transcriptomes from *D*. *variabilis* (data not shown). Based on the current evidence in ticks and mites, we cannot conclude that the methyltransferases in ticks are involved in the synthesis of methyl farnesoate; however, more work is needed to re-examine this question of whether ticks can synthesize methyl farnesoate. It is unlikely the tick methyl transferases are involved in the synthesis of JH in ticks, since there are multiple lines of evidence that ticks do not make or have JH and there is a lack of any biological effect when ticks are treated with JH (discussed earlier).

#### Farnesol oxidase in ticks

Two of the enzymes in the early part of the JH branch, farnesyl diphosphate pyrophosphatase and farnesal dehydrogenase (Fig 1), have not been characterized in insects and were not studied in our transcriptomes (Table 1) or in the *I*. *scapularis* genome [48]. At this juncture, there is no direct evidence the enzymes are present in ticks.

**Fig 6:**
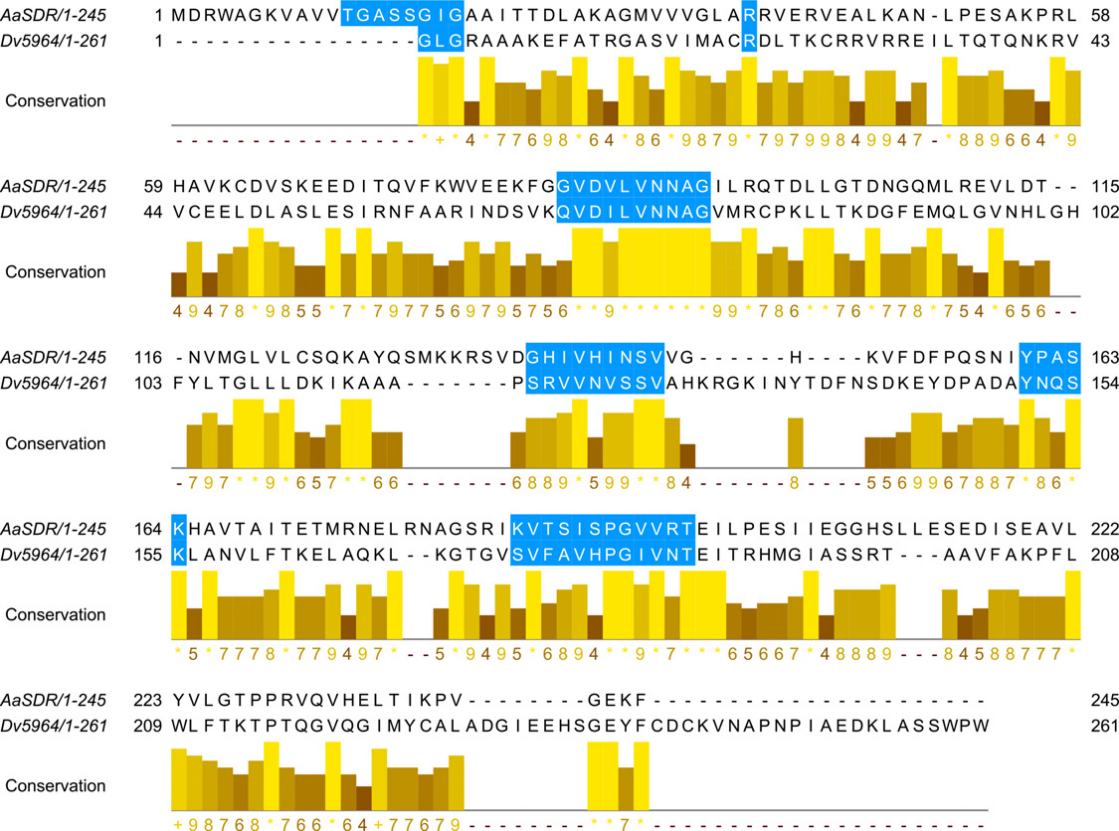
Farnesol dehydrogenase (AaSDR) from *Aedes aegypti* aligned with contig 5964 from the *D*. *variabilis* synganglion transcriptome. Highlighted residues on both alignments show the conserved motifs that place them in the SDR family and the subfamily cP2. Below the sequence alignment is the conservation panel which is measured as a numerical index (9–0) reflecting the conservation of physicochemical properties in the alignment. * (asterisk) denotes the highest identity score (identical residues in all species) followed by a score of 9 for the next most conserved group of residues containing substitutions by amino acids included in the same physicochemical class as described by Livingstone and Barton [52].

**Fig 7:**
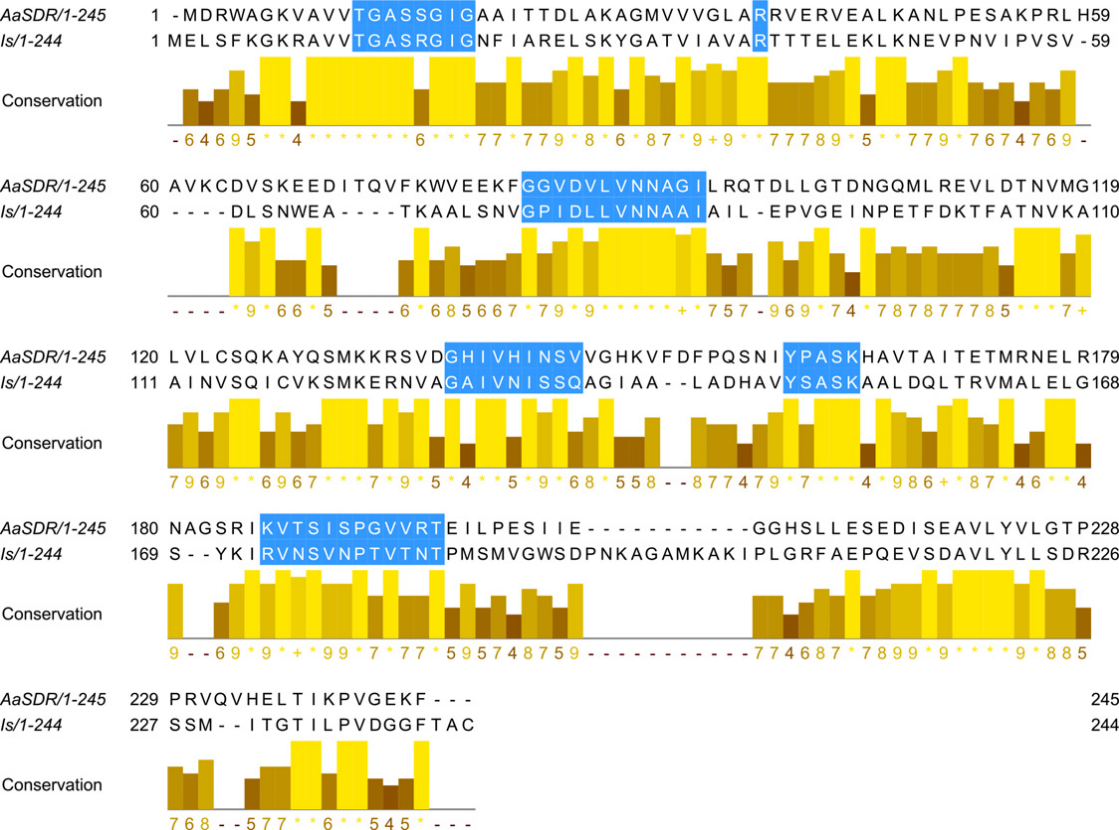
Farnesol dehydrogenase (AaSDR) from *Aedes aegypti* aligned with sequence EEC12752 from the *I*. *scapularis* genome. Highlighted residues on both alignments show the conserved motifs that place them in the SDR family and the subfamily cP2. Below the sequence alignment is the conservation panel, which is measured as a numerical index (9–0) reflecting the conservation of physicochemical properties in the alignment. * (asterisk) denotes the highest identity score (identical residues in all species), followed by a score of 9 for the next most conserved group of residues containing substitutions by amino acids included in the same physicochemical class as described by Livingstone and Barton [52].

Short-chain dehydrogenases are needed for the conversion of farnesol to farnesal (a farnesol oxidase) and then from fanesal to farnesoic acid (a farnesal dehydrogenase) (Fig 1). Mayoral et al. [63] characterized the amino acid sequence and function of an NADP+-dependent farnesol dehydrogenase (AaSDR), a farnesol oxidase, in the corpora allata of the mosquito, *Aedes aegypti*, which was shown to transform farnesol into farnesal. The AaSDRs have several conserved motifs that placed them in the SDR family of proteins and in the subfamily cP2, and orthologues have been found in another mosquito species, *Anopheles gambiae*, where they shared 61–67% identity with the *A*. *aegypti* AaSDR. Table 3 shows the BLASTp matches for a putative tick synganglion farnesol oxidase to the top three matches based on e-values in insects which were annotated as farnesol oxidases and to the functionally validated farnesol oxidase (AsSDR) in *A*. *aegypti* (shown in bold). For *D*. *variabilis*, the e-value was e-12 for the mosquito that was functionally proven as a farnesol oxidase with the top other putative farnesol oxidase matches ranging from e-102 to e-99 and for *I*. *scapularis*, was e-21 for the *A*. *aegypti* farnesol oxidase with top other matches ranging from e-102 to e-99. No matches were found for the soft tick. We reverse BLASTed the AaSDR sequence from *Aedes aegypti* against our *D*. *variabilis* synganglion transcriptome and found orthologues that matched the mosquito AaSDR sequence. The *D*. *variabilis* contig 5964 (KT602362) shared 24% identity and an e-value of 2e-9 with AaSDR. Based on the alignment in Fig 6, it shared several conserved motifs with AaSDR, but not all the motifs could be found due to the short contig length. From BLASTp searches conducted on the *I*. *scapularis* genome using the *A*. *aegypt*i farnesol oxidase, we found 79 dehydrogenases that shared 23%-30% identities with AaSDR (e-values ranging from e-21 to 0.001). We aligned the top match (EEC12752) from the *I*. *scapularis* genome with the *A*. *aegypti* AaSDR (Fig 7), and we found they shared 33% identity, with an e-value of 4e-22, and all the conserved motifs were present (Fig 7). It appears from both our transcriptomic studies in two hard tick species, *D*. *variabilis* and *I*. *scapularis*, and an analysis of the *I*. *scapularis* genome, ticks have the enzyme farnesol oxidase which is involved in the conversion of farnesol to farnesal. A direct functional analysis will be needed in the future to confirm this finding. Based on this discovery, a reasonable hypothesis is that the presence of farnesol oxidase argues for also the presence of farnesyl diphosphate pyrophosphatase (Fig 1) since the latter is responsible for the synthesis of the substrate, farnesol. It is also possible that a farnesal dehydrogenase is present to make farnesoic acid based on some evidence of methyl transferases in ticks and mites and the report of methyl farnesoate in mites. However, the evidence is conflicting that supports the presence of insect JHAMTs in ticks or that ticks can synthesize methyl farnesoate as already discussed. Our data based on the presence of a farnesol oxidase is that at least the early parts of the JH branch but not JH is present in ticks, and more work will be needed to understand what JH precursors in the JH branch are synthesized by ticks.

#### Model for the regulation of adult reproduction in ticks and a role for the mevalonate-farnesal pathway

**Fig 8:**
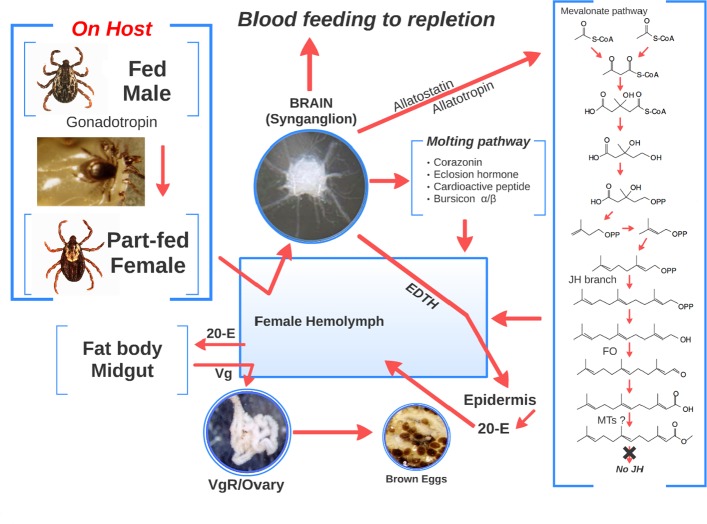
Model for the endocrine regulation of vitellogenesis in *D*. *variabilis* and the potential role of the mevalonate-farnesal pathway. Abbreviations in Fig: EDTH, hypothesized epidermal trophic hormone; Vg, vitellogenin; VgR, Vg receptor; 20-E, 20-hydroxyecdysone.

The most complete understanding of the endocrine regulation of tick development is associated with female reproduction, and the most advanced understanding of this process is in the American dog tick, *D*. *variabilis*. Fig 8 is a model summarizing our current understanding of this process in *D*. *variabilis* and the potential role of the mevalonate-farnesal pathway in reproduction. In the initial steps for reproduction, both males and females must find a host together. The females feed to a part-fed condition, stop feeding and remain attached. Once the males fully feed, they detach from the host, find the part-fed female, mount the female and then insert their mouthparts into the female genital pore. This stimulates the production of the spermatophore and along with gonadotropin is transferred to the female genital tract [1]. The transfer initiates the synganglion to release epidermal trophic hormone (EDTH), stimulates rapid engorgement in the female to the replete (fully fed) condition, initiates synthesis of the insect molting neuropeptides (the tick function is unknown), and release of allatostatins and allatotropins (which may stimulate or inhibit the mevalonate-farnesal pathway; Fig 8); these peptides can also have other function in insects. EDTH initiates the production of ecdysteroids by the epidermis, which results in the synthesis of VgR in the ovaries and Vg in the fat body and midgut; Vg is then secreted into the hemolymph. Vg moves into developing oocytes via VgR-receptor mediated endocytosis and is deposited as vitellin.

It appears that the enzymes in the mevalonate pathway are present in adult synganglia in at least the three tick species examined, *D*. *variabilis*, *I*. *scapularis* and *O*. *turicata*. There was no evidence of the P450 family CYP15A1 in either our transcriptomes or in the genome of *I*. *scapularis* which in insects adds the C10,11 epoxide to make JH. Along with the lack of biochemical evidence for JH in ticks and lack of JH biological activity in ticks, it appears ticks do not make JH. However, we provide evidence that an earlier part of the JH branch is present for the synthesis of farnesal from farnesol by the enzyme farnesol oxidase. Methyltransferases were also found in the synganglia, but they lacked the JH or farnesoic acid binding motif found in insects, and biochemical studies did not show ticks could synthesize methyl farnesoate or had methyl farnesoate in their hemolymph. We hypothesize that ticks synthesize JH precursors found in the JH branch at least at the level of farnesal but the presence of methyl farnesoate is equivocal and more work is needed. It is likely that the mevalonate-farnesal pathway has some regulatory role in adult development potentially being regulated by neuropeptides that in insects regulate JH biosynthesis, allatotropins and allatostatins, and which are also found in adult ticks. The exact role of the mevalonate-farnesal pathway is unknown and the products from this pathway have not yet been identified in ticks.
